# Insulin Syringe: A Gimmick in Pediatric Dentistry

**DOI:** 10.5005/jp-journals-10005-1458

**Published:** 2017-02-27

**Authors:** Gurpreet Kour, Updesh Masih, Chanchal Singh, Manvi Srivastava, Priti Yadav, Jagriti Kushwah

**Affiliations:** 1 Student, Department of Pedodontics and Preventive Dentistry, K.D. Dental College & Hospital, Mathura, Uttar Pradesh, India; 2 Professor, Department of Pedodontics and Preventive Dentistry, K.D. Dental College & Hospital, Mathura, Uttar Pradesh, India; 3 Professor and Head, Department of Pedodontics and Preventive Dentistry, K.D. Dental College & Hospital, Mathura, Uttar Pradesh, India; 4 Student, Department of Pedodontics and Preventive Dentistry, K.D. Dental College & Hospital, Mathura, Uttar Pradesh, India; 5 Student, Department of Pedodontics and Preventive Dentistry, K.D. Dental College & Hospital, Mathura, Uttar Pradesh, India; 6 Student, Department of Pedodontics and Preventive Dentistry, K.D. Dental College & Hospital, Mathura, Uttar Pradesh, India

**Keywords:** Insulin syringe, Local anesthesia, Sound, eye, motor scale, Wong-Bakers faces rating scale.

## Abstract

**Aim:**

The management of pain and anxiety in dentistry encompasses a number of procedural issues, including the delivery of anesthetic solution. One of the most important ways to manage the behavior of children is pain control. Trypanophobia is very common among dental patients and the most important goal of guidelines on behavior guidance for pediatric dental patient is to ease fear and anxiety in dental procedures in children. For the stated reasons, the purpose of the present study was to record child’s pain sensation both objectively and subjectively while receiving dental local anesthesia using conventional syringes and diabetic needles.

**Materials and methods:**

Twenty children of age group 6 to 12 years undergoing routine dental procedures participated in the study. Every child acted as one’s own control, while receiving treatment on the opposite side of the same arch. Each patient was randomly assigned to receive the injection either with conventional syringe or diabetic needle for the first visit, while the injection with the other needle was administered during the second visit. Rating scales were used for objective and subjective evaluations.

**Results:**

Statistical analysis of the measurements were made using Wilcoxon signed U test and Mann-Whitney U test which showed the mean sound, eye, motor (SEM) score difference using insulin syringe. The outcome was statistically significant when compared using the mean ranks between male and female patients with that of control group.

**Conclusion:**

It can be concluded that diabetic syringes exhibit clinical advantage and its use in pediatric dentistry for local anesthetics (LA) infiltration can prove beneficial.

**How to cite this article:** Kour G, Masih U, Singh C, Srivastava M, Yadav P, Kushwah J. Insulin Syringe: A Gimmick in Pediatric Dentistry. Int J Clin Pediatr Dent 2017;10(4):319-323.

## INTRODUCTION

As quoted by Dean Koontz, “Pain can be endured and defeated only if it is embraced. Denied or feared, it grows in perception if not in reality.” Pain is a complex and multidimensional construct that involves sensory, emotional, and cognitive processes.^1^ A very important part of dentistry is pain control. The most difficult aspect of patient management that can be a barrier to good care and treatment is fear-related behavior.^2^

Patients who fear dental treatment can induce anxiety and harm the smooth delivery of dental care.^3^ Not just the pain and discomfort, the prospect of injection and just looking at a syringe can provoke anxiety particularly in children.^4^

Most widely used drugs in dentistry are LA.^1^ In surgical and dental procedures, they prevent nociception. The only perceived painful part of dental procedures is the injection of LA.^2^ The field of dental medicine has always been trying to create a painless experience for the patients. Anesthetic needle injection attributes to the fear of pain, which has been a problem in providing appropriate dental care.^2,5,6^ In an attempt to improve patient comfort during dental anesthetic administration, smaller gauge needles, slow computer-regulated administration, distraction techniques, vibrating devices, and topical agents (refrigerants and anesthetics) have been used.

The major issues in delivering dental treatment to children are anxiety and phobia. So reducing the anxiety level even before giving a LA injection is necessary, especially in children and this can be done by using a syringe, which is smaller in size, colorful, and less frightening than the usual conventional syringes used.

**Fig. 1:**
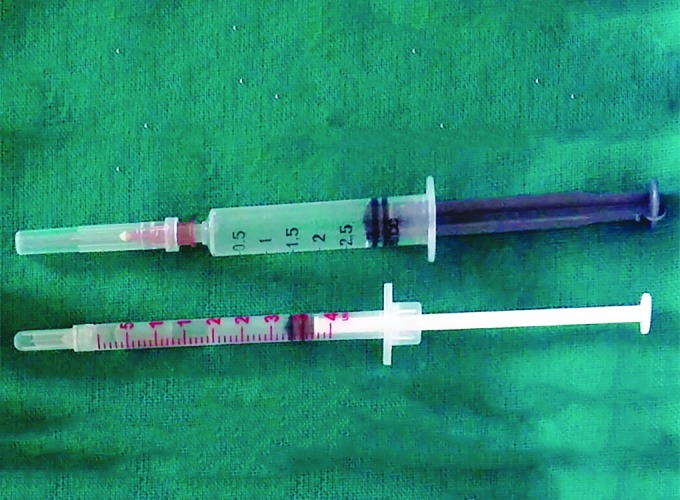
Conventional and insulin syringe

Therefore, the purpose of this study was to evaluate pain perception in children, while providing LA with 26 gauge conventional syringe and 30 gauge insulin syringe (Fig. 1).

## MATERIALS AND METHODS

The present *in vivo* study was undertaken in the Department of Pedodontics and Preventive Dentistry in K.D. Dental College & Hospital, Mathura, Uttar Pradesh, India, to compare the pain perception of children to LA using two different syringe designs.

Twenty children who met the inclusion criteria were selected for participation in this study.

### Inclusion Criteria

- Children from 6 to 12 years of age with treatment needs in two different quadrants either in maxillary or mandibular arch.
- A minimum of two clinical appointments of similar operative procedures on both sides of the same jaw preceded by LA injection.
- Children who demonstrated positive or definitely positive behavior during pretreatment evaluation (ranking 3 or 4 in the Frankl scale).
- Children having their first dental visit.

### Exclusion Criteria

Children with emergency treatment needs, such as abscess, cellulitis and space infection, and those who needed premedication for receiving dental treatment. All parents were informed about treatment procedures and an informed consent was obtained. The study was performed using two types of syringe designs. Local anesthetic solution used was 2% lidocaine and 1:100,000 epinephrine. Every child acted as one’s own control, while receiving treatment on the opposite side of the same arch. Patients were randomly assigned to receive the injection either with a conventional syringe or insulin syringe for the first visit, while the injection with the other syringe was administered during the second visit. Objective and subjective evaluations were done using two rating scales.

### Objective Evaluation

For objective evaluation during the injection procedure, the response of the child was noticed with SEM scale designed by Wright et al in 1991.^7^

### Subjective Evaluation

Immediately after injections, children were asked to complete the Wong-Bakers faces rating scale (FRS) for subjective evaluation of pain perception after the injection. Verbal instructions were given to the child on how to utilize the FRS. The values for this scale range between 0 and 5, where 0 is no hurt and 5 is hurt very much. The data were collected and analyzed using Statistical Package for Social Sciences version 17.0 for Windows. The level of statistical significance was set as 95% (p = 0.05).

The objective and subjective behavioral parameters were evaluated by Wilcoxon signed rank test and Mann-Whitney U test.

## RESULTS

The mean pain score using FRS scale for 26 gauge conventional syringe was found to be 3.25, whereas mean pain score for 30 gauge insulin syringe was found to be 1.35 (Table 1). The mean difference found was statistically significant (p = 0.05). The mean SEM score in patients receiving LA with conventional syringe was found to be 2.47 and for patients receiving LA with insulin syringe was 1.48. The mean difference was statistically significant.

**Fig. 2:**
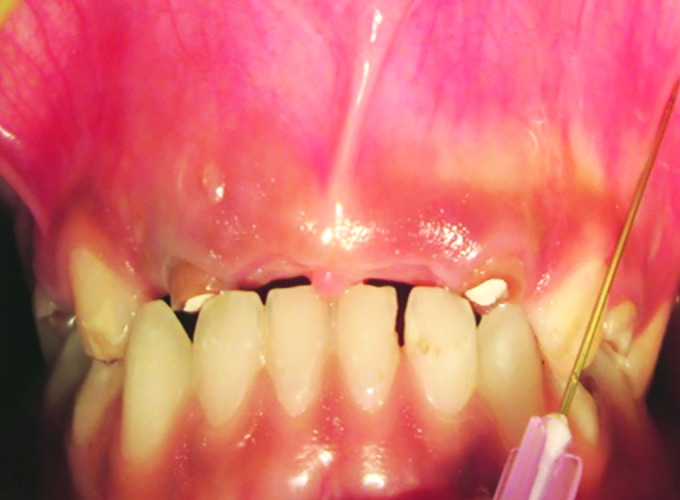
Local anesthetics administration using conventional syringe

**Fig. 3:**
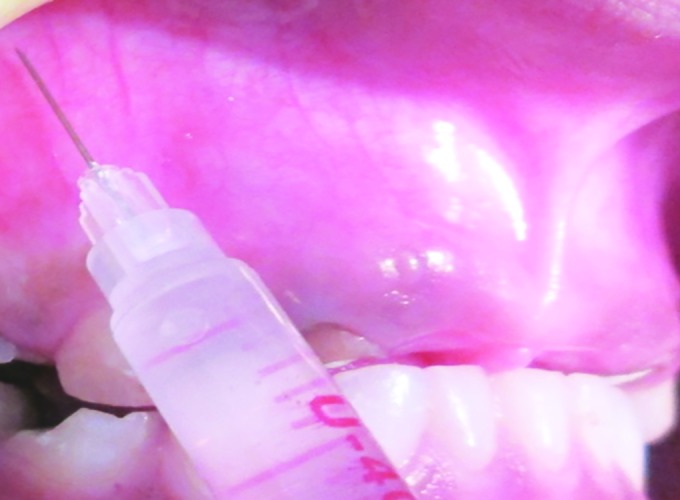
Local anesthetics administration using insulin syringe

The mean pain score in male patients using conventional syringe was 2.0 and score using insulin syringe was 1.20. The difference was statistically significant (Table 2). The mean SEM scores in male patients receiving LA conventional and insulin syringe were 2.12 and 1.24 respectively. The difference was statistically significant (Figs 2 and 3).

The mean pain score in female patients using conventional syringe was 3.40 and score using insulin syringe was 1.40 (Table 3). The difference was statistically significant. The mean SEM scores in female patients receiving LA with conventional and insulin syringe were 2.59 and 1.55 respectively. The difference was statistically significant.

**Table 1:** **Table 1:** Descriptive analysis—mean pain score

*Pairs*		*Variable*		*n*		*Mean*		*SD*		*Wilcoxon signed rank test (Z)*		*p-value*		*NS/S*	
Pair 1		IS-FRS		20		1.35		0.49		–3.976		0		S	
		CS-FRS		20		3.25		0.97						NS	
Pair 2		IS-SEM		20		1.48		0.34		–3.956		0		S	
		CS-SEM		20		2.47		0.58						NS	

**Table 2:** **Table 2:** Descriptive analysis (male group)

*Pairs*		*Variable*		*n*		*Mean*		*SD*		*Wilcoxon signed rank test (Z)*		*p-value*		*NS/S*	
Pair 1		IS-FRS		5		1.20		0.45		–2.070		0.038		S	
		CS-FRS		5		2.80		0.84						NS	
Pair 2		IS-SEM		5		1.24		0.13		–2.030		0.042		S	
		CS-SEM		5		2.12		0.52						NS	

**Table 3:** **Table 3:** Descriptive analysis (female group)

*Pairs*		*Variable*		*n*		*Mean*		*SD*		*Wilcoxon signed rank test (Z)*		*p-value*		*NS/S*	
Pair 1		IS-FRS		15		1.40		0.51		–3.450		0.001		S	
		CS-FRS		15		3.40		0.99						NS	
Pair 2		IS-SEM		15		1.55		0.35		–3.450		0.001		S	
		CS-SEM		15		2.59		0.57						NS	

**Table 4:** **Table 4:** Mann-Whitney U test

		*Gender*		*n*		*Mean rank*		*Sum of ranks*		*Mann-Whitney U score*		*p-value*		*NS/S*	
IS-FRS		Male		5		9		45		30		0.429		NS	
		Female		15		11		165							
IS-SEM		Male		5		5.8		29		14		0.023		S	
		Female		15		12.0667		181							
CS-FRS		Male		5		7.9		39.5		24.5		0.236		NS	
		Female		15		11.3667		170.5							
CS-SEM		Male		5		6.4		32		17		0.066		NS	
		Female		15		11.8667		178							

The mean SEM score difference using insulin syringe was statistically significant when comparing the mean ranks between male and female children using Mann-Whitney U test (Table 4). The mean SEM score ranks using conventional syringe for local infiltration for males was 6.4 and for females 11.8, which was not statistically significant. The mean FRS pain score differences using conventional and insulin syringes for local infiltration of LA between male and female child patients were not statistically significant.

## DISCUSSION

The present study was undertaken to evaluate and compare the pain perception using two different syringe designs with different needle gauges while giving LA using local infiltration technique. Dental treatment demands a good response of a child patient and his cooperative behavior.^4^ Any measure used to potentially minimize the pain during dental treatment can help in reducing the anxiety and fear of our child patient.

Behavioral management, distraction techniques, topical anesthetic agents prior to injections have been suggested to reduce pain during injection.^4^ To influence the objective behavior of the child, insulin syringes (U-40) were used in this study.

Infiltration technique has been used in this study because of various factors like direct vision of practitioner on it, less depth penetration of needle, less technical errors, less amounts of anesthetic solution, easier application, limited anesthesia of soft-tissues outside the operation field, and shorter duration of being anesthetized and might be used as an alternative to block.^1,8,9^

Donohue et al^10^ compared the effectiveness of infiltration technique with block technique and concluded that mandibular infiltration as a possible alternative to mandibular block anesthesia in young children.

Jones et al^11^ in a study on 308 patients, inferior dental nerve blocks were rated significantly more painful than buccal infiltrations. The visual analog pain scale was found to be unsuitable for use by children under 7 years of age, and keeping it in view, the age group was selected for this study.

Jung et al^12^ evaluated the efficacy of block and infiltration injections anesthetizing mandibular first molars and concluded faster appearance of anesthesia with infiltration injection compared to block with same efficacy.

Dental needles are available in three lengths: Long (32 mm), short (20 mm), and ultrashort (10 mm). Needle gauges range from size 23 to 30. Needle breakage is a rare occurrence and its primary cause is weakening the needle due to bending it before insertion into the soft tissues and patient movement after the needle is inserted.^1^

Short needles may be used for any injection in which the thickness of soft tissue is less than 20 mm. Any 23- through 30-gauge needle may be used for intraoral injections, since blood can be aspirated through all of them. Aspiration can be more difficult, however, when smaller gauge needles are used but an extra-short, 30-gauge is appropriate for infiltration injections.^1^

Various studies have been done and published by various authors to find the difference in pain perception using different needle gauges:

- Ghasemi et al^4^ concluded a significant difference concerning pain when 27 and 30 gauge needles were used and said that 30 gauge needle exhibited clinical advantage when used to give inferior alveolar nerve block in children. Ram et al^13^ also reported a significant difference concerning pain when mandibular nerve block was provided using 27 and 30 gauge needles.
- Cooley and Robison^14^ did a comparative evaluation of the 30-gauge dental needle and quoted that even under the extreme manipulations and stresses, the physical properties of these needles proved them to be tough, durable, and surprisingly resistant to breakage.
- Some authors like Fuller et al^15^ and Lehtinen^16^ have reported no significant differences in pain perception using different gauge needles.
- Brownbill et al^17^ compared inferior dental nerve block injections in child patients using 30-gauge and 25-gauge short needles, and it was concluded that 25- and 30-gauge needles do not differ significantly with respect to efficacy, pain, or aspiration.
- Asokan^18^ have concluded that the pain due to injection penetration may be controlled using thinner gauge needles.

The syringe to be used in first appointment was randomly selected to discard the effect of notion that the young patient brings to the initial dental experience, which might either facilitate or impede his adaptation to the stress.

No study to the best of our knowledge has been conducted till date, to check the influence of different syringe designs on the anxiety and fear psychosis of the pediatric patients, which in turn can influence the pain perception in our child patients while receiving the LA injection.

In this study, insulin syringes were compared with the conventional syringes. Insulin syringe with its miniature needle, bright color, and slim look appears like a toy to the child patient till our job of infiltration anesthesia is over. This study overwhelmingly justifies its use in pediatric patients as supported by the child. The use of insulin syringe for injecting LA solution also helps in curtailment of dental appointments in child patients as less time is required for convincing them to receive the injection and gaining their confidence as the syringe looks less menacing.

The calibrations in insulin syringe are marked at 0.025 mL intervals and so there is a controlled and fractionated administration of drug not requiring excessive force on the plunger, which can be monitored visually.^19^

The study justifies the use of diabetic syringe with 30-gauge needle to be used for the delivery of LA using infiltration technique in child patients because smaller gauge needle is less painful; the size and the color of the syringe is such that it does not scare the child patient, cost-effective, and the calibrations at 0.025 mL intervals in insulin syringe provide a drug delivery control, which in turn reduces the pain caused and there is less tissue distension, less chances of local ischemia and necrosis.

## CONCLUSION

It can be concluded that diabetic syringes exhibit clinical advantage and its use in pediatric dentistry for LA infiltration can prove beneficial for patients as well as for dental caregiver. There is scope of introducing toy syringes in market for the use by pediatric dentists.
